# The dynamic nature of cognition during wayfinding

**DOI:** 10.1016/j.jenvp.2008.02.006

**Published:** 2008-09

**Authors:** Hugo J. Spiers, Eleanor A. Maguire

**Affiliations:** Wellcome Trust Centre for Neuroimaging, Institute of Neurology, University College London, 12 Queen Square, London WC1N 3BG, UK

**Keywords:** Navigation, Virtual reality, Verbal reports, Planning, Spatial memory, Routes

## Abstract

Much of our day-to-day wayfinding behaviour takes place in familiar large-scale urban environments, yet there is a dearth of studies examining how wayfinding unfolds on a second-by-second basis in this context. Here we used a retrospective verbal report protocol, eye tracking and a highly accurate virtual reality simulation of a real city (London, UK) to examine this issue. Subjects, who were taxi drivers, were able to produce extremely detailed accounts of what they had been thinking during wayfinding, which were validated by independent eye-tracking data. There was a high degree of consistency in the types of thoughts across subjects, permitting classification into a number of distinct categories. Moreover, it was possible to quantify the number of thoughts in each category, their durations and temporal order. Detailed analysis of the verbal reports provided new insights into the processes and strategies involved, and highlighted a greater range of thoughts than has previously been reported in studies of wayfinding. By analysing the temporal order of thoughts it was possible to identify specific relationships between categories. Some of these relationships were predicted by current cognitive models of wayfinding, others were novel, thus shedding new light on how navigation unfolds in a busy city.

## 1 Introduction

Finding one's way in spatially extended environments is essential for survival and requires a wide range of cognitive abilities. Pre-eminent among these is the ability to make use of long-term spatial memory to guide wayfinding. An understanding of how we might mentally represent large-scale space has been gained from a variety of tests such as sketching maps, direction pointing and giving route descriptions (Chase, 1982; Garling & Garling, 1988; Giraudo & Peruch, 1988a, 1988b; Golledge, 1999; Pailhous 1970, 1984; Peruch, Giraudo, & Garling, 1989; Thorndyke & Hayes-Roth, 1982). These studies suggest that multiple, qualitatively different types of spatial representations can support wayfinding, including route knowledge (knowing the directions to turn at locations/landmarks) and survey-like knowledge (an integrated knowledge of the spatial relationships between locations/landmarks) (Siegel & White, 1975; Thorndyke & Hayes-Roth, 1982). It is thought that survey-like knowledge, also referred to as cognitive maps (Tolman, 1948), endows us with the ability to take detours and shortcuts in familiar environments.

In addition to the use of static tasks, such as sketching maps, examination of in situ ongoing behaviour during wayfinding has provided additional insights into the use of spatial representations, and identified factors affecting performance. Such factors include subjects’ spatial processing abilities, their familiarity with the environment, the density/salience of landmarks, and the layout of the environment (Foo, Warren, Duchon, & Tarr, 2005; Gillner & Mallot, 1998; Golledge, 1999; Holscher, Meilinger, Vrachliotis, Brosamle, & Knauff, 2007; Janzen, Schade, Katz, & Hermann, 2001; Newman et al., 2006; Thorndyke & Hayes-Roth, 1982; Wiener, Schnee, & Mallot, 2004). In particular, and of primary interest here, investigating how routes are chosen or planned has proved useful for identifying a number of wayfinding strategies (Conroy-Dalton, 2003; Golledge, 1995; Hochmair & Frank, 2002; Pailhous, 1970, 1984; Wiener & Mallot, 2003; Wiener et al., 2004). For example, Pailhous (1970, 1984) found that Parisian taxi drivers tended to make use of a familiar *primary network* of streets to facilitate navigation. By contrast, in another strategy known as the *least-angle* strategy, paths are chosen that minimize deviation from the angle pointing directly to the goal (Conroy-Dalton, 2003). Different again is the *fine-to-coarse* planning heuristic that argues routes are planned in fine detail in the currently occupied region, but only coarsely when planning navigation between regions (Wiener & Mallot, 2003). Finally, the *least-decision-load* strategy specifies that subjects will often choose the path with the least number of possible decision points (Wiener et al., 2004).

To account for this range of empirical findings, a number of cognitive models of wayfinding have been proposed (Garling, Book, & Lindberg, 1984; Kuipers, 1978; Kuipers, Tecuci, & Stankiewicz, 2003; Passini, 1981, 1984; Peponis, Zimring, & Choi, 1990; Stern & Portugali, 1999; Timpf, Volta, Pollock, & Egenhofer, 1992). All models distinguish between the processes of: (a) planning the route and (b) executing the plan, and generally contain a hierarchy of staged processes that unfold sequentially and iteratively during wayfinding. The models differ in the features they emphasize and situations they cover. Some deal with selecting the route to multiple destinations (Garling et al., 1984), others with navigating in unfamiliar environments (Garling et al., 1984; Peponis et al., 1990) or city streets (Garling et al., 1984; Kuipers, 1978; Kuipers et al., 2003; Timpf et al., 1992). For example, Kuipers’ models (Kuipers, 1978; Kuipers et al., 2003) build on the findings of Pailhous (1969, 1984) by using a primary network of roads to facilitate navigation. Timpf et al.'s (1992) model deals with the translation from route plans into the actions necessary to perform driving manoeuvres.

Whilst observing in situ wayfinding has generated empirical and theoretical advances, one significant limitation is that mere observation can make it difficult to determine the reasons or intentions behind some of the different actions performed. For example, stopping at a junction might be motivated by the need to reorient, look ahead, make a decision, or any number of other reasons. In order to dissociate such possibilities several studies have employed verbal report protocols (Chebat, Gelinas-Chebat, & Therrien, 2005; Dogu & Erkip, 2000; Gerber & Kwan, 1994; Holscher et al., 2007; Kato & Takeuchi, 2003; Passini, 1981, 1984; Titus & Everett, 1996). These protocols involve subjects either ‘thinking aloud’ during the task (concurrent verbal reports) or describing what they remember thinking during a structured interview after the experimental task (retrospective verbal reports) (Ericsson & Simon, 1980). The contents of the verbal reports are analysed with a pre-determined classification scheme usually developed from pilot studies. These classification schemes make use of commonalities in the statements contained in the verbal reports to derive a number of relevant ‘thought’ categories. Examining the categories and their contents can then be used to understand the cognitive task in question and potential strategies employed. By their nature, verbal report protocols can only give insight into the processes which subjects are aware of and are able to verbalize. Nevertheless, the reports gained can help provide evidence to distinguish between competing models seeking to explain cognition during a task (see Ericsson & Simon, 1980; Jack & Roepstorff, 2003).

Passini (1981, 1984, 1992) was the first to critically assess the wayfinding process with a verbal report protocol. His results, in agreement with other models, provided evidence for two core stages in wayfinding: formulating the route plan and executing the plan. Analysis of the verbal reports also identified a hierarchical organization in the route plans, with decisions at the top relating to over-arching goals, and decisions at the bottom related to sub-tasks. Route planning elements always contained two parts: a behaviour component (e.g. turn left) and a location/landmark specifier (e.g. at the central square). Thus, Passini argued that wayfinding could be conceptualized as a process in which route plans are initially set up and executed by actions at the appropriate place and time leading finally to the goal. In his model, plans generate expectancies to find particular locations/landmarks in order to perform the actions. These actions are triggered when a match occurs between a mental image of the expected locations/landmark and the experience of seeing it. When there is no such match a new plan must be formulated to solve the problem.

Since Passini's (1981, 1984, 1992) seminal studies, verbal reports been used to examine how a number of factors affect wayfinding cognition and strategy use. These factors have included the use of maps (Gerber & Kwan, 1994), subjects’ navigational aptitude (Holscher et al., 2007; Kato & Takeuchi, 2003), familiarity with the environment (Chebat et al., 2005; Holscher et al., 2007), the layout of the environment (Holscher et al., 2007) and wayfinding in the context of shopping (Chebat et al., 2005; Dogu & Erkip, 2000; Titus & Everett, 1996). Findings from these studies have generally agreed with Passini's (1981, 1984) model, and have helped to identify the use of a number of additional wayfinding strategies (Gerber & Kwan, 1994; Holscher et al., 2007; Kato & Takeuchi, 2003). For example, Holscher et al. (2007) found some subjects completely planned the whole route before setting off, while others would formulate only partial plans. The latter was found to be associated with poorer performance. Kato and Takeuchi (2003) found that some subjects were able to flexibly use a range of information to navigate, whereas others employed ineffective strategies, such as using parked cars as landmarks.

While these studies have shed some light on the cognitive processes during wayfinding, several important issues remain unexplored. The environments of the previous studies that used verbal reports were either indoors (Chebat et al., 2005; Dogu & Erkip, 2000; Holscher et al., 2007; Passini, 1981, 1984; Titus & Everett, 1996) or unfamiliar (Gerber & Kwan, 1994; Kato & Takeuchi, 2003). Thus, despite the fact that much of our daily wayfinding occurs in familiar outdoor environments, the cognitive processes involved in navigating them has yet to be examined. Prior studies have generally focused on decision making or strategy use, ignoring other components such as the visual processing of the environment or the entities within it. No study has examined how wayfinding unfolds on second-by-second basis in a large-scale environment. Appreciating the temporally fine-grained behaviour during navigation could give important insights into the underlying spatial representations and the wayfinding processes that act upon it. Related to this, quantitative examination of the temporal relationships between different types of thoughts during wayfinding has also been largely ignored. Finally, there has been little attempt to use the information in verbal reports to critically evaluate different cognitive models of wayfinding (Garling et al., 1984; Kuipers, 1978; Kuipers et al., 2003; Passini, 1981, 1984; Peponis et al., 1990; Stern & Portugali, 1999; Timpf et al., 1992).

Here we addressed these outstanding issues by using a retrospective verbal report protocol, eye tracking and a highly accurate virtual reality simulation of a real city (London, UK) to explore the second-by-second thoughts accompanying wayfinding. We also used the information obtained from the verbal reports to examine temporal contingencies in the thoughts, and to evaluate cognitive models of wayfinding.

## 2 Materials and methods

### 2.1 Participants

Twenty healthy right-handed male licensed London taxi drivers participated in the experiment (mean age: 49.8 years, SD: 8.5 years, range: 27–59 years). Taxi drivers were used in order to investigate wayfinding in subjects familiar with the environment, and to provide a consistent level of performance. The average time spent working as a taxi driver was 18.3 years (SD: 10.9 years, range: 1–38 years). All had either lived in London their entire lives or for the vast majority of it and were naïve to the stimuli used in the experiment. All subjects gave informed written consent to participation in accordance with the local research ethics committee.

### 2.2 The virtual environment

**Fig. 1:**
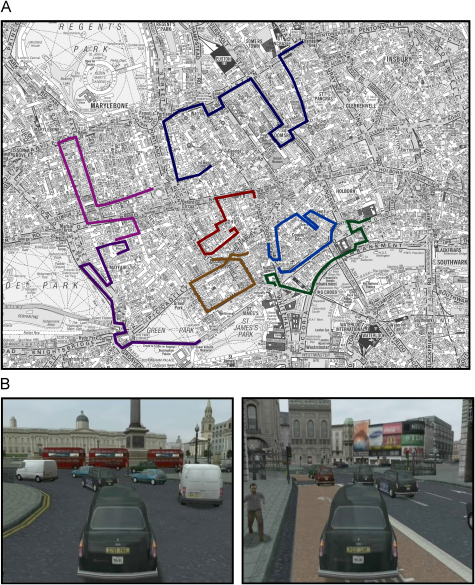
Virtual London (UK). Panel (A) shows a map of the region of the simulation of London that was used in the navigation task (not all the minor streets shown were included in the video game). Reproduced by permission of Geographers’ A-Z Map Co. Ltd. © Crown Copyright 2005. All rights reserved. Licence number 100017302. Coloured lines indicate examples of typical routes driven by subjects to each of seven destinations during the navigation task. Panel (B) shows example views from within the video game ‘The Getaway’ © 2002 Sony Computer Entertainment Europe. Left image shows a view at Trafalgar Square, right image shows a view at Piccadilly Circus. These images are reproduced with the kind permission of Sony Computer Entertainment Europe.

The video game ‘The Getaway’ (© Sony Computer Entertainment Europe, 2002) run on a Sony Playstation2 (© Sony Computer Games Inc.) was used to present subjects with a ground-level interactive first person perspective view of a simulation of central London, UK (see Fig. 1, and Spiers & Maguire, 2006a for a movie of navigation through the environment). While desk-top virtual reality simulations have their limitations, such as a lack of peripheral and binocular visual information, there are numerous advantages to using VR. In the current study use of VR London made it possible to assess navigation in a familiar yet complex large-scale environment in a controlled way that simply would not be possible with in situ navigation in this city. In addition, use of VR permitted recording of subjects’ performance and allowed for collection of eye-tracking data (for more on VR versus real environments see Morganti, Carassa, & Geminani, 2007; Peruch & Gaunet, 1998; Richardson, Montello, & Hegarty, 1999; Stanton, Wilson, & Foreman, 2002). In the virtual London game used in this experiment over ∼110 km (∼70 miles) of driveable roads have been accurately recreated from Ordinance Survey map data, covering approximately 50 km^2^ (∼20 square miles) of the city centre. The game designers decided to truly recreate the city and a large team of photographers walked the streets of central London for two years recording details of the city. Buildings, shops, the one-way systems, working traffic lights, the busy London traffic and an abundance of Londoners going about their business are all included. The ‘Free Roaming’ mode of the game was used, permitting free navigation with the normal game scenarios suspended. Subjects moved through the environment in a virtual London taxicab controlled using a game controller, consisting of two joysticks providing analogue control of acceleration, braking and steering left and right. The virtual taxi had a higher degree of controllability than many of the other vehicles in the game, and its low top virtual speed could be maintained easily. Even slight contact between a subject's vehicle and other vehicles could result in a crash which would prematurely terminate the experiment. Therefore a ‘cheat’ modification was employed using Action Replay Max software (© Datel Design and Development Ltd., 2003) which prevented such crashes. Subjects were instructed to drive ‘legally’ as they would in actual London. To assess the ecological validity of the virtual simulation, during the pilot testing, subjects were asked to comment on the similarity of the game to the real city, and their sense of ‘presence’. All remarked that Central London (where the experiment was set) in particular was highly accurate and detailed with a high level of presence. Following the experiment proper subjects were asked how similar the simulation and task was their everyday experience. All of the taxi drivers confirmed that the game was very reminiscent of their experience of driving in central London.

### 2.3 Pre-test training and familiarization

Two weeks prior to testing, subjects were given over 2 h of practice with the game controls by asking them to navigate to various locations in areas of environment not used in the experimental task. To avoid waiting for long periods at red traffic lights, subjects were familiarized with treating all traffic lights as green, but were otherwise required to comply with all other road traffic regulations in the UK. Thirty minutes before testing subjects were again given further practice in an area not used in experimental tasks. During this practice session subjects were trained to respond to a set of recorded customers’ requests to take them to destinations in London. Prior to testing, subjects were told the locations they would be starting from in the experimental tasks, but not the order.

### 2.4 Experimental task

The experimental tasks took place during functional magnetic resonance imaging (fMRI) brain scanning. Details of the scanning parameters and related fMRI analyses can be found in Spiers and Maguire (2006a, 2006b, 2007a, 2007b). In the experimental task, navigation was tested in blocks where subjects responded to customers’ requests (heard via head phones) by delivering them to their destinations. During each block one route was tested. Seven routes were included which were (in order): Kings Cross to the Middlesex Hospital (initially to Guy's Hospital), Trafalgar Square to the Royal Courts of Justice (initially to the Old Bailey), St. Giles Circus to Peter Street in Soho (initially to Paddington Station), St. Martin's Place to Leicester Square (initially to Covent Garden), Piccadilly Circus to Leicester Square (initially to St. James's Square), Buckingham Palace to the American Embassy (initially to Shepard's Market) and the American Embassy to Cavendish Square (initially to Manchester Square). When the game came on the screen, subjects were given between 3 and 5 s to orient themselves in the environment. Following this they heard a customer request a destination (mean duration 2.0 s). For all routes, at some point during navigation the subjects heard customers request a change of destination (mean duration 3.0 s). For three of the routes an additional request to avoid a location or go via a location was made by the customer (mean duration 3.7 s). Requests for a change of destination or requests to go via another location occurred at time points that varied along each route and were unknown to the subject but predetermined by the experimenters. Two subjects completed only four routes, in one case due to discomfort, the other due to a technical problem. Each block of navigation ended when either the subject reached the destination or when a predetermined period of time elapsed. The maximum time taken to get to each of the destinations was between 3 and 6 min (mean: 3.8 min, SD: 1.1 min). Each block of navigation was separated by a period of rest in which the subjects viewed a blank white screen for 60 s. Total mean time for the experimental task was 31 min 35 s (SD: 4 min 9 s).

### 2.5 Video recording

In order to debrief subjects and create an independent record of eye tracking, two videos were recorded during the experimental task. Video output from the Playstation 2 was split into three ways: (1) to a projector presenting stimuli to the subject on a screen in the MRI scanner (view angle of 27.6°), (2) to a VHS video recorder for debriefing and (3) to a video mixer to create an eye-tracking video. Video output going into the video mixer was combined with camera footage of a stopwatch manually synchronized with the time stamp on the debriefing video. Gaze position cross hairs collected via an ASL504LRO infra-red eye-tracker (Applied Science Laboratories, Bedford, MA) were overlaid onto the video sent to the mixed video recording in 14 subjects. Accurately calibrated eye-gaze tracking was achieved in nine subjects.

### 2.6 Verbal report protocol

Immediately after the experimental task the subjects were taken to a separate room where they were given a surprise debriefing with a verbal report protocol (Ericsson & Simon, 1980). In this debriefing, subjects watched the video of their performance during the experimental task. They were carefully instructed to describe what they remembered thinking, step-by-step, during their original performance. The interview proceeded at a pace determined by the subject, with the video being paused and rewound by the interviewer where necessary to capture the details provided by the subject. A new copy of the original video was recorded during the interview with the voices of the subject and interviewer collected by a microphone overlaid. In accordance with the methods described by Ericsson and Simon (1980), the interviewer followed a predetermined protocol during the interview. A subject's report was interrupted as little as possible, the interviewer intervening only to improve the subject's specification of the onset and duration of thoughts where possible, and on occasion where clarification was required to later aid analysis. The mean duration of the collection of the verbal reports was 108.9 min (SD: 16.9 min).

### 2.7 Analysis of the retrospective verbal reports

**Fig. 2:**
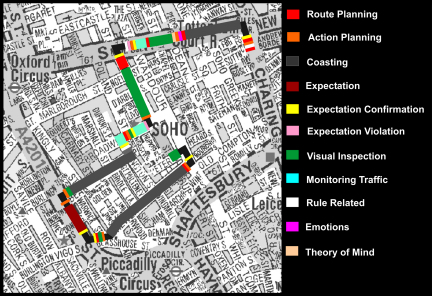
The route driven by an example subject (subject 3) from Charing Cross Road to Peter Street in Soho is shown segmented into different colour-coded thought categories (see explanatory key on the right-hand side) derived from the subject's verbal report. Note that turning, not in the key, is shown in black on the route. The route is indirect due to the one-way road system in Soho.

Anonymized audio information from the verbal report interviews was transcribed by a professional transcription agency who were blind to the purpose of the experiment. By comparing the transcript with the time stamp from the original performance video, information about the timing of the thoughts was incorporated into the transcripts and any errors or unclear statements rectified. Each statement in the transcript was then classified into one of a set of categories, and where appropriate sub-categories (see Table 1), and its onset and duration recorded to create a segmented timeline of every subject's experience of every route (see example in Fig. 2). Unambiguous categories were predetermined by analysis of common repeated statements in the verbal reports of four subjects who took part in an in-depth pilot study prior to the main experiment. This process was also guided by findings from previous studies employing verbal report protocols and wayfinding tasks (Chebat et al., 2005; Dogu & Erkip, 2000; Gerber & Kwan, 1994; Holscher et al., 2007; Kato & Takeuchi, 2003; Passini, 1981, 1984; Titus & Everett, 1996). The pilot study also served to create a guide to aid classification and generate a protocol for the initial collection of the verbal reports. The independent eye-tracking video was used to aid the identification of onsets and durations where the subjects reported looking at a feature in the environment, and so served as an external measure to validate the procedure. Environmental features consisted of static structures (e.g. buildings and statues) and moving objects (e.g. vehicles and pedestrians). For each report of looking at a feature in the environment, the eye-tracking video corresponding to that time point was examined to determine when, to the nearest second, a saccade to the feature was made and how long fixation of the feature occurred in seconds. To assess the degree to which the verbal reports could be reliably categorized, a second experimenter classified the verbal reports contained in 36 random extracts. Of note, it was not just taxi drivers who were able to produce detailed retrospective verbal reports. Several non-taxi driver subjects who navigated in the game through areas of the city they were familiar with were able to produce reports as accurate as those of the taxi drivers.

### 2.8 Analysis of the temporal precedence of thought categories

In order to understand the temporal relationships between categories, we examined whether the thoughts belonging to some categories preceded the thoughts belonging to other categories more or less often than would be expected by chance. This was done by constructing a contingency table for each subject which was a transition matrix of all the possible pairings of categories. This table contained the number of *observed* occurrences in which events of each category directly preceded the events of each other category. For each pair we also calculated the number of occurrences that would be *expected* by chance. This was done by multiplying together the number of events in each category and then dividing by the total number of events across all categories. We then collapsed across subjects by summing the number of observed occurrences and summing the number of expected occurrences in each subject's table to create one table. A *χ*^2^ test was used to test whether the number of observed occurrences provided a good fit to the number of expected occurrences in this table, with the degrees of freedom=(number of columns −1)×(number of rows −1). If a significant result was obtained, a *χ*^2^ test was used for each category to test whether events from this category preceded events in each of the other categories more or less often than was expected from chance. If a significant result was obtained in any of these *χ*^2^ tests, sign tests were then used to probe individual category pairs. Sign tests examined whether there were significantly more subjects for whom the number of observed occurrences was greater or less than the number of expected occurrences.

## 3 Results

Aspects of the findings from this rich and flexible data set involving fMRI have been reported elsewhere (Spiers & Maguire, 2006a, 2006b, 2007a, 2007b). We now report new analyses focused on the separate issue of understanding cognitive process during wayfinding, and how they inform models of wayfinding.

### 3.1 Behavioural performance and verbal reports

All subjects completed the task successfully with a mean of 94% (SD: 9%) of their routes being efficient. An efficient route was one where the subject moved continually closer to the goal destination given the constraints of London's one-way system and occasional obstructed streets that were included in the game (see Fig. 1). Using mapping software (Map24(UK): http://www.uk.map24.com) it was possible to determine that subjects travelled a mean total distance of 16.9 virtual km (SD: 3.4) during the experimental task.

Subjects were able to produce detailed accounts of what they had been thinking during wayfinding. There was a high degree of consistency in the types of thoughts across the 20 subjects. Reviewing the transcriptions of all subjects, and aided by a classification guide developed during the piloting phase, 12,484 thoughts were classified into distinct categories (see Table 1 for examples). Not only was the level of detail in the retrospective verbal reports very high, subjects reviewing the video of their wayfinding performance were quite clear about when exactly they had experienced particular thoughts and in what order. This enabled a complete specification of the wayfinding experience in terms of the onsets, durations and temporal order of thoughts for each subject. The precision of the timings was further tested using the independent eye-tracking data acquired during the experimental task. In those subjects with accurate calibration, 94% (SD: 6%) of reports of looking at a feature in virtual London whilst navigating were corroborated by a saccade to its location at (or very near, ±2 s) the time retrospectively reported. In addition, agreement was found between the two experimenters for 93% of classifications arising from the random sample of statements that were dual-classified (*κ*=0.91).

### 3.2 Overview of how wayfinding unfolds in a familiar city

Our analysis of the verbal reports permitted a detailed breakdown of how wayfinding unfolds in a familiar large city (see Table 1, Fig. 2). In summary, we initially plan our route to a destination; en route we might adjust our plan because of new opportunities or obstructed paths. Sometimes we plan our route only to an intermediate point and once reached, we then fill in the rest of the route plan. Often within a familiar environment we are almost on automatic pilot, ‘coasting’ along without thinking. We also set up expectations, waiting to see the next junction or a landmark to confirm we are on the right track, occasionally inspecting the city around us as we travel through it. We monitor the surrounding traffic to achieve safe passage to our destination, and have to be able to plan our own actions, such as staying in a traffic lane or changing lanes. Sometimes our emotional state might change, or we pause to consider the thoughts of the people we encounter. It is notable how recognisable the above aspects are to our everyday experience of urban wayfinding, and yet many of them have gone largely undocumented. What one truly appreciates from the retrospective verbal reports is how, second by second, the underlying cognition can change (Fig. 2). We now consider each of the thought categories in turn, and provide qualitative characterization of their salient features.

### 3.3 Route planning

**Fig. 3:**
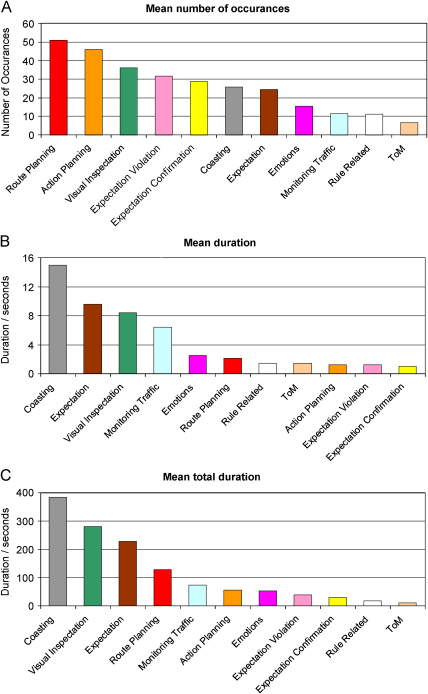
Frequency of thoughts and their durations across subjects for each category: (A) the mean number of occurrences for each thought category, (B) the mean duration of each period for each thought category and (C) the mean total duration across all routes of each thought category.

**Fig. 4:**
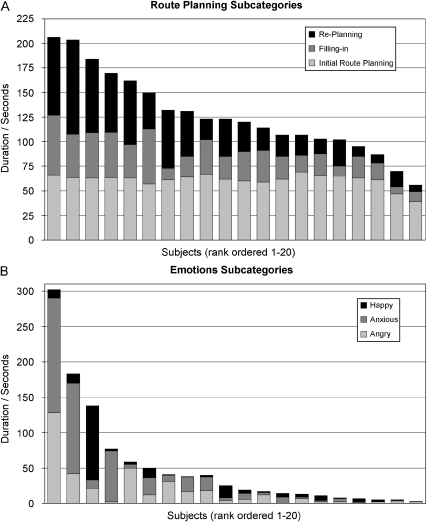
Route planning and emotions: sub-categories. (A) The total duration of each sub-category of route planning for each subject (rank ordered by total number of events). (B) The total duration of each sub-category of emotion for each subject (rank ordered by total number of events).

Route planning is a crucial stage in wayfinding and it occurred more frequently than any other category (see Fig. 3). In Appendix A we provide detailed extracts from verbal reports describing route planning. It was possible to identify three sub-categories of route planning: initial route planning, filling-in and re-planning (see Table 1). In the current study, initial route planning was driven by either the customers’ requests at the start of each route or the customers’ requests for a change in destination en route. No differences were discernable in the verbal reports of route planning following these two causes of initial planning. Across subjects, the occurrences and durations of both filling-in and re-planning were similar (see Fig. 4).

Examination of the verbal reports shows that initial route planning involved a number of distinct stages (see Table 1 and Appendix A). In the first instance, the destination's location must be retrieved. After this, the direction to the location must be determined. This process was reported on by most subjects. A few subjects’ descriptions were in terms of cardinal directions (see Extract 2, Table 1), but most described it in an egocentric reference frame (see Extract 4, Table 1 and Extract 7, Appendix A). For many subjects, determining the direction is the most important aspect of the process (see Extracts 6 and 8, Appendix A). The next stage of route planning involves street selection, and was more diverse across routes and individuals. This ranged from only selecting the next street, to completely specifying the streets to the destination. Some subjects reported thinking of the street names, while others did not (see Extracts 10–13, Appendix A). Most often the streets selected were described starting with the nearest street and ending with streets close to the destination (see Extract 1, Table 1 and Extract 5, Appendix A), but occasionally there were reports of starting from the destination and working backwards (see Extract 21, Appendix A). Street selection appeared to be affected by the distance or number of streets to the goal destination. In addition, when selecting the streets subjects often accounted for the one-way road traffic system. The time of day and day of the week were also important considerations as they related to road congestion and the obstruction of streets due to street markets. Strategies were varied, one of the most common being to initially plan a route that would end up facing in a direction towards the destination, and then filling in the rest of the route (see Extracts 7 and 8, Appendix A). Another strategy was to plan a route to a particular region or access point and then plan the next part of the route from there (see Extracts 23 and 24, Appendix A). When choosing between options, some subjects reported attempting to keep it simple rather than choosing intricate routes (see Extract 6, Table 1). Some subjects reported planning a few different route options in case of problems en route, and also reported re-assessing the route plan after it had been selected (see Extracts 17, 25 and 26, Appendix A). A few subjects described imagining driving down the route at high speed in their ‘mind's eye’ as part of a checking process (see Extracts 17, 18 and 22, Appendix A). Reports of imagery were not confined to ground-level views, but also very occasionally included aerial views (see Extract 19, Appendix A), but no imagery of a map was reported.

The verbal reports revealed that route planning rarely stops after the initial plan. En route subjects often filled-in a plan for next stage of the journey or re-planned part of the route. These two other types of route planning were subject to similar considerations and strategies involved in initial route planning, but typically fewer streets and directions were specified. Filling-in was typically precipitated by having finally reached a street facing towards the destination, reaching the end of a previously planned route segment, or reaching a new region. Often the filling-in commenced when the subject had finished turning into the street they planned to reach, rather than at the moment of spotting the street (see Extract 5, Table 1). Re-planning often occurred because a street was obstructed (see Extract 7, Table 1). Several subjects commented that this was quite a common occurrence in London. Re-planning was also caused by subjects spontaneously realizing there was a better route available, or very occasionally by accidentally passing the street they intended to use (see Extract 8, Table 1). There was a lack of mental imagery in descriptions of filling-in and re-planning.

### 3.4 Action planning, monitoring traffic and rule related thoughts

Once route planning is complete we need to make the appropriate actions to reach our goal. Many actions were made without subjects reporting any prior planning, such as keeping the vehicle in a straight line. However, other actions were reported to be preceded by a plan. These action plans were distinguished from route plans by the fact that they specified a single action rather than a sequence of actions, and the action was to be executed at a location currently in view. Action planning was the second most common category after route planning (see Fig. 3), indicating that thinking about future events comprises a considerable portion of the wayfinding experience. Many action plans involved thoughts about turning into a street that was currently in view and part of the route plan. Planned actions also included: changing lanes, making U-turns and manoeuvring round moving vehicles. Action planning involving other traffic also involved monitoring the movements of these vehicles. Subjects also reported monitoring the movement of vehicles on other occasions, such as when looking out for a street, to see if it was accessible. Buses were often a concern for the subjects, since they provided the greatest risk of collision (see Extract 30, Table 1). Like route planning, action planning was also affected by road traffic rules, such as the one-way streets and road markings (see Table 1). Often these related to inhibiting particular actions, such as complying with ‘no stopping’ signs (see Extract 36, Table 1).

### 3.5 Coasting

Whilst route and action planning are the thought categories that occurred most frequently, examining total amount of time subjects spent in each cognitive state revealed a different story. For a good deal of the time spent wayfinding in this familiar city subjects reported ‘coasting’ along without any directed thoughts (see Fig. 3). This typically occurred on long stretches of road where subjects knew they did not need to make any further decisions (see Extracts 12 and 15, Table 1). Several subjects used the terms ‘automatic pilot’ or ‘switched off’ to describe the experience.

### 3.6 Visual inspection, expectation and confirmation or violation of expectations

During any wayfinding excursion there are moments when we need to look around to acquire information about the environment. Four distinct categories related to this were identifiable in the verbal reports. Some periods of wayfinding were spent visually inspecting fixed features in the environment, while on other occasions inspection occurred with the expectation of seeing a specific feature not yet in view (see Table 1 and Fig. 3). Visual inspection could occur out of a general interest in the local environment (e.g. Extracts 26, 27 and 28, Table 1) or in order to extract useful information from it, for example, from road signs (e.g. Extract 29, Table 1). Expectation periods, by contrast, involved looking out for the next turning to execute the route plan or a landmark to confirm the subject was on the right path. Expectation periods often commenced after turning into a street, but also after certain amount of time had elapsed driving down a street (see Extract 18, Table 1). Often a period of expectation would be followed by confirmation that the feature sought had been spotted. Alternatively on occasion the expectation was violated when, for example, the street was blocked off or the expected feature was visually altered (e.g. scaffolding covering up a landmark).

### 3.7 Theory of mind and emotions

In addition to planning, coasting and visual processing, subjects also reported thinking about the thoughts and intentions of other individuals (known as ‘theory of mind’) and also reported changes in their own emotional state. Theory of mind thoughts related to moving agents (such as pedestrians and fellow road users), the customers, and the experimenters. Some thoughts were concerned with actions made, or the intentions of the customer (see Table 1), or thinking about what others might think of their own behaviour (see Spiers & Maguire, 2006b for more details). Subjects also reported changes in their own emotional state which fell into three sub-categories: happy, anxious and angry (see Table 1). Anxious and angry thoughts dominated, with some subjects identifiably more ‘emotional’ than others (see Fig. 4). A wide variety of causes could induce the reported transitions into emotional states, such as a road being blocked off (angry), getting to the destination (happy) and nearly crashing (anxious), see Table 1.

### 3.8 Temporal precedence

A temporal precedence analysis was used to test for any temporal relationships between the thought categories. We found that the events in some categories preceded the events in other categories either more or less often than was expected by chance (*χ*^2^=2402.9, df=90, *p*<0.001). Subsequent analyses revealed that the observed temporal relationships were significantly different from the pattern predicted by chance for all categories except theory of mind. These results and the results of post hoc sign tests are reported in Table 2. To summarize, occurrences of expectation confirmation and expectation violation were significantly more likely to be preceded by expectation. A significantly greater number of action planning events were preceded by monitoring traffic, rule-related thoughts and expectation confirmation than would occur by chance. In contrast, route planning was only preceded by one thought category, expectation violation. Both action planning and route planning frequently preceded coasting periods. Monitoring traffic often preceded expectation confirmation and often occurred after expectation and expectation confirmation periods. Several categories occurred less frequently after others than would be predicted by chance, and generally follow a natural logic. For example, expectation confirmation thoughts were not likely to be immediately followed by periods of expectation violation.

## 4 Discussion

In this study we used a retrospective verbal report protocol, eye tracking and a highly accurate virtual reality simulation of a real familiar city to explore cognition during wayfinding on a second-by-second basis. By classifying statements in subjects’ verbal reports into a number of pre-determined thought categories we were able to characterize their wayfinding experience in terms of the diversity, frequency, duration and temporal order of thoughts. The high degree of correspondence between verbal reports of looking at features in the environment and independent eye-tracking measurements provided support for the validity of the verbal reports. A statistical analysis of temporal precedence of thought categories allowed us to identify their temporal relationships. Combining this information with a detailed qualitiative examination of the verbal reports, we now evaluate the ability of extant cognitive models to capture the rich wayfinding process we have revealed, as well as comparing our findings with those from previous wayfinding studies that employed verbal report protocols.

Cognitive models of wayfinding share a number of core features. Our finding of distinct route planning and action planning processes, and that these two categories occurred most frequently, lends support to the notion that these two stages form a central core of wayfinding cognition (Garling et al., 1984; Kuipers, 1978; Kuipers et al., 2003; Passini, 1981, 1984; Peponis et al., 1990; Stern & Portugali, 1999; Timpf et al., 1992). Examination of the verbal reports of initial planning revealed that it was composed of three sequential stages: (1) retrieving the location of the destination, (2) determining the direction to the destination and (3) retrieval/selection of the streets to form the route. Sequential processing of information as part of initial route planning is a common feature in the cognitive models. However, some models generally ignore the first two stages, focusing instead on the selection of places and paths (e.g. Kuipers, 1978; Kuipers et al., 2003; Timpf et al., 1992). By contrast, the models of Garling et al. (1984) and Passini (1992, 1984) both involve retrieval of information prior to planning. However, the stages in Garling et al.'s (1984) model differ from those we found. In their model the first stage involves the retrieval of *all* the relevant information (destination and paths), and subsequent stages deal with the selection of places and the paths linking them. This difference may relate to the fact that Garling et al's (1984) model was devised to deal with the problem of navigating to a sequence of multiple destinations, where the order in which the destinations can be visited needs planning. In this context it is useful to retrieve the locations of all destinations and possible paths first in order to decide which paths are optimal. Our data suggest that when single destinations are considered the location of the destination is retrieved first and then the subsequent stages occur subsequently.

Another feature of route planning under emphasized in many models is the requirement to determine the direction to the destination. Our subjects often noted that this was a very salient component of initial route planning. This makes sense if we consider that wayfinding will fail without it, and in some situations knowing only the direction may be sufficient to reach the goal (see Spiers & Maguire, 2007b for further discussion). The lack of emphasis of getting the right direction in verbal reports of route planning in previous studies may relate to the use of indoor environments, where the next waypoint may be visible from the outset.

The final core stage of initial planning, retrieving/selecting the streets, contributed most to subjects’ route planning descriptions and it also features most prominently in wayfinding models (Garling et al., 1984; Kuipers, 1978; Kuipers et al., 2003; Passini, 1981, 1984; Peponis et al., 1990; Stern & Portugali, 1999; Timpf et al., 1992). The models of Kuipers (1978, 2003) and Timpf et al. (1992) consider route planning as the construction of a set of instructions for movement between places along paths in a cognitive map stored in memory. In agreement with such models, the verbal reports often contained a specification of the paths (streets) and directions (left, right) necessary to reach the destination. Similar to the models, the travel instructions were mostly specified starting near the current location and progress, street by street, towards the goal (Garling et al., 1984; Kuipers, 1978; Kuipers et al., 2003; Passini, 1981, 1984; Timpf et al., 1992). The fact that many routes were not completely specified is also consistent with the partial planning suggested in several models (e.g. Garling et al., 1984; Passini, 1981, 1984) and similar observations in previous studies (Holscher et al., 2007; Passini, 1981, 1984). Passini's (1981, 1984) model draws the distinction between this process of retrieving the map of the environment to select the streets and the direct retrieval of stored instructions for very familiar routes. Our data generally support this distinction, in that for some routes no streets were specified and subjects stated they just knew where to go. This also agrees with previous reports of the rapid ‘automatic’ route choices made by experienced taxi drivers (Chase, 1982; Pailhous, 1970, 1984). However, our data also serve to highlight that even in familiar environments planning routes to some destinations can be very detailed, with multiple factors considered and strategies used.

A number of strategies for aiding route planning have been identified in previous studies and incorporated into some cognitive models (e.g. Garling et al., 1984; Kuipers, 1978; Kuipers et al., 2003; Timpf et al., 1992). By strategy we mean the use of a particular heuristic or switching of behaviour that is not a requirement for the task but improves performance or frees up cognitive resources. Some strategies we identified could be related to those previously described, while others were novel and not previously reported. In Passini's (1981, 1984) model decisions are hierarchical, organized with over arching goals at the top-level and sub-goals beneath. This was not a dominant feature of the route plans of our subjects. This difference may relate to the fact that Passini's model is based the verbal reports of navigation inside buildings (see also Holscher et al., 2007). However, one strategy reported did fit this approach: initially planning a route to end up facing in the direction toward the destination before planning the full route. The layout of the environment and the route requirements have also been suggested to influence route planning strategies (Conroy-Dalton, 2003; Holscher et al., 2007; Wiener & Mallot, 2003; Wiener et al., 2004). The least-decision-load strategy (Wiener et al., 2004) was observed in descriptions where subjects opted for the simpler route when choosing between two similar route options. The fine-to-coarse heuristic (Wiener & Mallot, 2003) was also evident in the reports of subjects who first selected the region in which the destination lay and then planned the route to that region, and the partial planning was for routes covering large distances. Strategies we identified that are not obviously specified in models included re-checking the route once it has been selected, using ground-level mental imagery of moving through the streets to see if it is correct, and the planning of multiple route options in case of problems. The use of mental imagery in navigation has been reported in some studies (Gerber & Kwan, 1994; Passini, 1984), but not others (e.g. Chebat et al., 2005). Gerber and Kwan's (1994) observation that some subjects visualized the next five streets ahead is consistent with the reports from some of our subjects. Like taxi drivers from Paris and Chicago, London taxi drivers do not appear to use bird's eye imagery of a map for planning routes (Chase, 1982; Pailhous, 1970, 1984). Thus, if survey-like representations (Siegel & White, 1975; Thorndyke & Hayes-Roth, 1982; Tolman, 1948) support wayfinding on some routes in familiar environments, they do not take the form of mental images of maps.

Another strategy, deduced from the study of Parisian taxi drivers, is the use of primary street networks to facilitate wayfinding (Pailhous, 1970, 1984). Chase (1982) found no evidence for such a strategy in studies of Chicago taxi drivers. Similarly in our data there is no evidence for an explicit use of this strategy. However, the representation may be more subtle. Recently, a retired taxi driver with damage to his hippocampus bilaterally (patient TT) was tested on his navigation in the virtual simulation of London used in the current study (Maguire, Nannery, & Spiers, 2006). Patient TT could only navigate accurately when the destinations could be reached using predominantly the main arterial ‘A’ roads. Thus, it may be that when navigational ability is compromised, a primary coarse network of London's major streets is retained and helps to support residual navigational ability.

All models distinguish between the need to form an initial route plan and make further plans en route. Most focus on re-planning caused by changes to the environment, such as a street being blocked off. Our temporal precedence analysis showed that indeed route planning events were more often preceded by expectation violation events than would be predicted by chance. Re-planning could also be driven by subjects spontaneously realizing there was a better option or by missing a turning, additional reasons not considered in the models. A few models draw the distinction between filling-in and re-planning (Passini, 1981, 1984; Wiener & Mallot, 2003). Our finding that filling-in often occurs after the subject had entered a new street rather than as soon as this street was spotted does not feature in any model. Such a delay may relate to the subjects cognitive resources being taken up with making an appropriate action (e.g. changing lane) to turn into the street.

Several models highlight the importance of the link between the observation of an expected landmark/view (expectation confirmation) and a cue to perform a particular action (action planning) (Garling et al., 1984; Kuipers, 1978; Kuipers et al., 2003; Stern and Portugali, 1999; Timpf et al., 1992). Our temporal precedence analysis provides direct support for this link. It also revealed that in the context of driving it is not just static features of the environment that can initiate this process. We found monitoring traffic often preceded expectation confirmations. Examining the verbal reports suggested this was mostly caused by monitoring the traffic on the road ahead to detect the next street in the route plan and then monitoring their movements after the street had been detected to assess its accessibility. This relationship between monitoring traffic and navigational cues was not predicted by models involving vehicle driving (Kuipers, 1978; Kuipers et al., 2003; Timpf et al., 1992).

The existence of periods of expectation during navigation replicates similar observations in from previous studies employing verbal reports (Chebat et al., 2005; Holscher et al., 2007; Passini 1981, 1984). However, unlike Passini's verbal reports, there was no indication from our subjects that they were holding a mental image in mind during these periods. Our results revealed that expectation was not the only state that subjects were occupied by during wayfinding. The most common state to be in was in fact coasting along, not thinking directed thoughts. Given its prevalence, it is surprising that this category was not identified in previous studies examining verbal reports in wayfinding tasks (Chebat et al., 2005; Dogu & Erkip, 2000; Gerber & Kwan, 1994; Holscher et al., 2007; Kato & Takeuchi, 2003; Passini, 1981, 1984; Titus & Everett, 1996). One likely reason for this is that many of the previous studies used concurrent ‘think aloud’ verbal report protocols. In ‘think aloud’ procedures, coasting periods would have been observed as gaps between utterances and thus not necessarily classified.

Another category missing from the descriptions in previous studies and models are thoughts relating to visual inspection, where subjects reported looking at a particular fixed feature in the environment. After coasting, visual inspection accounted for more time during the task than the other thought categories. Some models include the need to update information from signs or environmental features (Garling et al., 1984; Passini, 1981, 1984), which would fall into this category. However, this only represents one possible reason for visual inspection. Many of the statements involved looking at features such as statues, shops and large buildings simply out of curiosity, rather than to gain specific information. Thus, visual inspection may relate partly to keeping oriented in the environment, but also to generally looking at features out of interest.

Whilst visual processing of the environment has received little attention in the models, other aspects of cognition we identified in our study are completely absent in the models. The categories emotions and theory of mind were reported by all subjects. While these may not be necessary for successful wayfinding, they are not irrelevant to it. For instance, it is often useful to be able to predict/interpret the actions of other people, since they may know something important about the environment. For example observing someone reversing out of a street might lead you to wonder why they chose to take that action—perhaps the street beyond is blocked. Wayfinding can be stressful, particularly when it involves driving in a large city where road rage is a too frequent occurrence. The observation that angry and anxious thoughts dominate the emotional experience confirms that this was the case for many of the subjects in the current experiment.

To summarize, our findings agree with many components of current models of wayfinding, but also reveal new features not captured by them. The sequential and hierarchical nature of route planning outlined in models was evident in our data. As were the distinctions between route planning and action planning, and between initial route planning and spontaneous route planning. We observed several previously described route planning strategies, including the least-decision-load strategy (Wiener et al., 2004) and the fine-to-coarse planning heuristic (Wiener & Mallot, 2003). Several models predicted the sequence of thoughts: expectation, expectation confirmation and action planning, which were related to carrying out the route plan (Garling et al., 1984; Kuipers, 1978; Kuipers et al., 2003; Passini, 1981, 1984). Aspects of our data not captured in the models include certain components of route planning, monitoring traffic to detect the next waypoint, and the under-emphasis of visual processing and other cognitive states during wayfinding. With regard to route planning, extant models fail to reflect the importance of determining the direction to the destination. We found no evidence for the use of a primary network of roads to facilitate route planning (Kuipers et al., 2003; Pailhous, 1970; Pailhous 1984), but instead we found a number of other strategies including planning the route to a location facing towards the destination and then planning the rest of the route. Overall, our results indicate that wayfinding involves much more than simply planning, and carrying out plans. It can evoke a range of emotions, spark interest in the surrounding environment and lead one to consider the thoughts of fellow wayfinders.

In conclusion, much of our day-to-day wayfinding behaviour takes place in familiar urban environments. By combining interactive virtual reality and retrospective verbal reports, the complexity and dynamic nature of the cognition behind wayfinding in a well-known busy city has been revealed. Our findings lend some support to extant models of wayfinding, but also suggest that they might require revision to account for the diversity and temporal order of thoughts, as well as additional wayfinding strategies. In the future, further use and development of verbal reports are recommended as a potentially powerful means to interpret wayfinding behaviour.

## Figures and Tables

**Table:** 

*Planning familiar routes*
Extract 1: Sub. 17
Because I’ve worked the area [*Soho*] so much, it's automatic. If you get regular things it's automatic, you don’t really have to think about it, you know it better than your own front room. When you go in your front room you don’t think: “I’ll go and switch the television on, where is it?”
Extract 2: Sub. 1
Sometimes when you’re out on the road, you do it [*route planning*] instantly without even thinking
Extract 3: Sub. 2
Well, it's an instinct and familiarity really. You’ve done it so often that it doesn’t really test your brain. Obviously some do. It's only when you get sort of difficult ones, or possibly places you’ve not been for a long while. And then you’re actually thinking all the way along. I had one last week that I’ve not been through in 20 years and the names came back and I still found it
Extract 4: Sub. 3
Do you want to know one of the worst things about driving a cab? You have a lot of time to think. Not about the job because that comes instinctively, to think about other things. People say “oh I’m too busy at work, I haven’t been able to think about that” I have all day. I spend more time thinking about other things, this [*route planning*] clicks in very quickly
Extract 5: Sub. 6
It's virtually instant… As you change the path of that vehicle you’ve made your mind up, basically, for 90% of the route. And that one's only three streets in my book. Strand, Fleet Street, Ludgate Hill, I’m there
*Determining the direction*
Extract 6: Sub. 2
Basically, you’ve got to be like a… a weather vane. You’ve actually got to be facing in the right direction, and that's the art of it, really. It is an instantaneous reaction as to where you’re making for, really. Because you can’t sit there wondering, getting a book out wondering whether you’ve got to turn around or do a U-turn or left. It's got to be an instant reaction
Extract 7: Sub. 17
You can actually think too hard, and if you think too hard—you can’t think. So what you do is you say well, the Middlesex Hospital is back that way, so you don’t panic the brain, you leave the brain free and then what you do is you turn it and point it in the right direction and then you start sorting it out
Extract 8: Sub. 18
The important thing when I start is to be heading in the right direction and then that gives you enough time to sort of think as to where you go. So sometimes it's made up as you go along as well
Extract 9: Sub. 1
As I said, you tend to follow your nose and trust your instinct because you know that you’re going in the general direction
*Verbalizing street names*
Extract 10:
Experimenter
And are you thinking the street names?
Sub. 6
Yes, yes, yes. Definitely, yes
Extract 11:
Experimenter
Do you think through the names of the streets?
Sub. 20
Not verbally, it's quicker than that. You haven’t got time to verbalize
Extract 12: Sub. 2
I don’t think, oh, I’ll turn left Duncannon Street into the Strand, left into the Aldwych, left into, er, Catherine Street. However, that's how you do it at the Carriage Office [*examination centre for the licence*] because that's the only way they know that you know where you are going. You don’t obviously think that way when you’re driving, you just sort of do it. It's just automatic, really
Extract 13: Sub. 3
I’m sure I was much more worried when I first started and I would have probably remembered the names of all the streets I was going down and would be going down, whereas now it's far more automatic
*Filling-in the next stage of the journey*
Extract 14: Sub. 3
I know I’m going in the right direction and I’ won’t make a decision until I get closer towards the end. That's how it works
Extract 15: Sub. 17
What I’m actually doing is allowing myself time—this is very delicate, I never knew it was as delicate as this—but what you’re actually doing is: instead of taking the whole route in one go, you’re take a safe step forward to the next one to reappraize where you’re going to go and then it gives you time to think and to relax. And then what you do is you take the next stage stepping stone, but by then you’ve always got it, you’ve had enough time to zoom it in
Extract 16: Sub. 18
Once you’ve got the basic plan in your head and you just fill in the detail as you go along
*Mental imagery*
Extract 17: Sub. 17
And that's all visual in my mind. When I do it here, I zigzag visually, so I do a fast forward. The nearest you can get to it is very, very… less than a second, very, very fast I go de-de-de-de-de that fits. So in my mind I’m going very fast visually and I’m seeing the streets, not from a plan bird's eye view, but just forward fast, left, right and it's done. It's done as quick as that, but it's visual. It's checking—yes, that's it, that’ll work
Extract 18: Sub. 6
You see it like… it's like a Schumacher run [Formula *one motor car race*]. Yes. It's just like that. And you see it in your mind. Jun, jun, jun, It's done in seconds. Because of the nature of the job, you’ve also got to concentrate on controlling the vehicle and putting it in the right location to do that journey
Extract 19: Sub. 17
I’ve got an over-patched picture of Peter Street. It sounds daft, but I don’t view it from ground level, it was slightly up and I could see the whole area as though I was about 50 foot up. And I saw Peter Street, I saw the market and I knew I had to get down to Peter Street
Extract 20:
Experimenter
Were you imagining driving all the way down to the Old Bailey?
Sub. 17
No, it's too easy. It's just the Strand, Fleet Street, do a left. I didn’t have to with that one
Extract 21: Sub. 18
I’d pictured the hospital up just off Great Portland Street then I brought it back to the Marylebone Road, then I turned round
Extract 22: Sub. 20
I think you do think in images. Maybe you fill in details and overlay it with words and things later because, I mean, your brain works in images. What I think, when you say a place like Royal Courts of Justice, I’m thinking what street's it in, that's the first thing…That's how everyone sort of learns it initially, so Royal Courts of Justice is in the Strand. That's what I’m thinking initially and then I’m thinking both, what road it's in or what it looks like. So it could be either option. The Royal Courts of Justice, I probably thought the Strand; then I pictured it. If it doesn’t snap in to perspective straightaway you normally need a keyword, the two things go together to pick up a memory somewhere. When it works, instantly then you’ve got like a snap, if you like, flashing ziggly, zaggly line and you can fill in the detail as you go along. And it's more or less right
*Navigating by region*
Extract 23: Sub. 1
There are various points in London where there are only one way in and one way out and when you do the knowledge, even though I did it 20 years ago, you learn those ways in and they stick
Extract 24: Sub. 15
You take a region and head for the region first and then sort of branch out
*Considering options and re-assessing*
Extract 25: Sub. 18
I pretty much know what I’m going to do and what I’m going to come across and also just bearing in mind that some roads are closed. I’ve got at the back of my mind maybe another alternative route just in case of… the roads, the roads are closed. I remember someone did say to me once when I first started he said just have a couple of options just in case there is traffic, you can’t do a specific turn or something
Extract 26: Sub. 18
Obviously once you’ve got the route, then you double check it several times in your head. Because sometimes, you know, you’ve got it in place and you’re getting there and you think, no, hang on, that's completely wrong.

Statements in square brackets and italics provide additional information to clarify what the preceding statements refer to. Statements in parentheses and italics provide information about a subject's behaviour.

**Table 1:** Category definitions and example extracts from verbal reports

*Route planning: initial*	Planning after a request from a customer to be taken to a specific destination
Extract 1	
Customer	Please could you take me to the Old Bailey?
Sub. 1	I thought, great, turn right, straight down the Strand, straight down Fleet Street and then left up to the Old Bailey
Experimenter	Did you picture the Old Bailey?
Sub. 1	No. I don’t think I did. I just know where it is
Extract 2	
Customer	Please could you take me to Guys’ Hospital?
Sub. 13	As soon as he said Guy's Hospital it's like I’m sitting here in the cab, I know Guy's Hospital is there (*subject points in the correct direction to Guy*’*s Hospital*). I know it's in that direction. So I know Guy's is now south east. In my body it's behind me and to the east and to the south. Straight away I can see… it's like I can see through all the buildings. It's like if I said to you picture from here to… the other room where we went, you can picture the whole surroundings, can’t you?
Experimenter	Yes
Sub. 13	And the door and the hallway. That's how it is with the roads
Extract 3	
Customer	Sorry, no, I need to get to Peter Street, the one off Wardour street
Sub. 3	I’m thinking *where the hell is it in Wardour street* (*emphatic tone*). So then, well I know instinctively I’ve got to turn left cos it's—I know where it isn’t. I know one little street that I’m not sure of… it must be that one, that was me instinct
Experimenter	Did you picture Wardour street?
Sub. 3	I had to picture the whole length of Wardour street in my head very quickly just knocking off the streets that I knew and then Peter Street was this little street. I kinda figured it might be that one cos I knew it wasn’t this one, this one, this one, or this one
Extract 4	
Customer	I’d like to go via Glasshouse Street, please
Sub. 14	Well, I knew that's just by Piccadilly Circus, which is over to our right. At this stage I don’t think I thought east or west. I knew it was like sort of two o’clock from here
Experimenter	Was there any plan?
Sub. 14	Well, I knew I was in a one way street so I just had to carry on
*Route planning: spontaneous—filling-in*	Planning the next stage of the journey
Extract 5	
Sub. 3	So as I turn then I think right now—okay when I get to the top there's a one-way system so yo’re forced left and right, left and right, there's a multitude of ways you can go from here so—but I know I’ve got to go to the end of the street because fundamentally I don’t want to take a left before that
Extract 6	
Sub. 7	I was going to use Brunswick Square and Guildford Street. Well, I wasn’t too sure whether to come a bit low with it and go Montague Street, Great Russell and then go up Earnshaw. That was a bit long winded I thought. Then I thought well we can get Judd Street and we can go down Tavistock Place. There was three different options came in my head
*Route planning: spontaneous—re-planning*	Altering the current route plan
Extract 7	
Sub. 12	And then I think hold on, there's another way. It's like I said before, there always seems to be an option comes in your way. Um, so I thought well, okay then, if I can turn right, then I’m going to go up through the back of there and across the church
Extract 8	
Sub. 9	Here, I’ve thought oh no, I’ve gone past Marlborough Gate, but I thought well, it doesn’t matter… I’ll just do Piccadilly
*Action planning*	Planning future movements with the vehicle
Extract 9	
Sub. 2	I just thought, well, I’ll go through there and once I turn right I’ll stay on the right hand-side
Extract 10	
Sub. 6	I’m thinking, I must get over to the right now
Extract 11	
Sub. 9	I’m just thinking about getting over onto the left hand lane to turn left
*Coasting*	Navigating automatically without any directed thoughts
Extract 12	
Sub. 1	You go into automatic pilot
Experimenter	Were you were in automatic pilot here?
Sub. 1	Once I’d seen the underpass, yes. Because everything is mapped out and I know which way I’m going
Extract 13	
Sub. 3	I’ve kinda switched off here, just keeping the car straight really. I use the term switched off, you don’t actually switch off, but you switch off thinking about your direction and maybe thinking about what's going on
Extract 14	
Sub. 11	It's as if someone else is doing it for you, all you are is a computer and you just program in and off it goes
Extract 15	
Sub. 20	I’ve got a dead straight run for quite a bit, so you don’t need to think much at all
*Expectation*	Looking out for the next expected environmental feature
Extract 16	
Sub. 1	Now I’m looking for the Euston underpass
Extract 17	
Sub. 2	I was expecting to see it… about now
Extract 18	
Sub. 6	Now I’m going to look out for it (*Mortimer street*)
Experimenter	What was it that told you to start looking out for it?
Sub. 6	I’m thinking I’ve traveled a long way along here now
Extract 19	
Sub. 21	Should be going downhill now, that's what I was thinking
*Expectation confirmation*	Detecting the presence of an expected environmental feature
Extract 20	
Sub. 1	And then all of a sudden… a little bit further on you go through these lights… and there it is. There's the cab shelter on the left-hand side
Extract 21	
Sub. 7	But then, that's it, I knew that was Glasshouse Street because that's the shape of it
Extract 22	
Sub. 9	And then when that yellow van comes up, yes, that's Orion House
*Expectation violation*	Detecting the absence of an expected environmental feature
Extract 23	
Sub. 6	I thought, oh, bloody hell, they’ve blocked it off
Extract 24	
Sub. 7	And then we come round here and then I got to about there and I thought *oh*, *no*, *please no* (*emphatic tone*). And then it was confirmed it was closed
Extract 25	
Sub. 14	Well, now I knew that I was coming up to Tottenham Court Road, I wanted to go straight on and here I saw that I couldn’t
*Visual inspection*	Visual inspection of an environmental feature
Extract 26	
Sub. 3	As I’m going along here, I thought oh look that's Dwight [*Statue of General Eisenhower*]
Extract 27	
Sub. 11	I was looking at it and I thought that's the American Embassy
Extract 28	
Sub. 17	As I turn, I’m looking at Boots [*chemist shop*] and the other shops
Extract 29	
Sub. 7	I’m just checking it [*the street*] and I could see the no entry arrow there
*Monitoring traffic*	Watching moving traffic in the environment
Extract 30	
Sub. 5	Well, the bus is a nuisance. You can’t see in front of it. It's in the way
Extract 31	
Sub. 7	I’m looking at the cabs over the other side in the bus lane
Extract 32	
Sub. 9	What I thought of here was: I’ll let that car go and I was very relieved that it accelerated away from me
*Theory of mind*	Thinking about other people's thoughts/intentions
Extract 33	
Sub. 11	I mean he [*the customer*] may be thinking I’m going to go down to Piccadilly Circus, back up Shaftsbury Avenue and in through that way, I don’t know, you know, or he may have got Glasshouse Street and Brewer Street mixed up
Extract 34	
Sub. 12	[*a car pulls out in front of him and the subject has to break to avoid a collision*] I thought what's he doing?.. Why would he do that? There's a line there, he's got to stop, you know, and that… I’ve got preference
*Rule related*	Thinking about the one-way system and road traffic rules
Extract 35	
Sub. 3	And then I realised, no it's one-way, I can’t go that way
Extract 36	
Sub. 11	And then there, I thought to myself: don’t park on the zigzags [*road markings indicating no stopping*]
Extract 37	
Sub. 9	I thought no, no, no, it's a one way street I won’t do that
*Emotions—happy*	Change in emotional state to being happy
Extract 38	
Sub. 6	I’m very happy with the way I’ve driven it now. I’m chuffed to bits
*Emotions—angry*	Change in emotional state to being angry
Extract 39	
Sub. 21	I was getting annoyed, I was actually getting annoyed
*Emotions—anxious*	Change in emotional state to being anxious
Extract 40	
Sub. 9	And I’m scared, like a horse, frightened by the railings

Statements in square brackets and italics provide additional information about the environment or people referred to by the subject. Statements in parentheses and italics provide additional information about the subject's gestures and voice tone.

**Table 2:** Results from the temporal precedence analysis

Category	Sign test *p*-value
*χ*^2^-score, *p*-value	
Route planning	
135.4, *p*<0.001	
Was *more* often followed by:	
Coasting	0.002
Rule related	0.004
Visual inspection	0.004
Was *not* often followed by:	
Expectation confirmation	0.001
Expectation violation	<0.001
Coasting	
75.3, *p*<0.001	
Was *more* often followed by:	
–	
Was *not* often followed by:	
Route planning	<0.001
Action planning	<0.001
Rule related	0.003
Expectation confirmation	0.021
Expectation violation	0.021
Action planning	
92.7, *p*<0.001	
Was *more* often followed by:	
Coasting	0.002
Was *not* often followed by:	
Expectation confirmation	0.003
Expectation violation	<0.001
Emotions	0.003
Visual inspection	0.008
Expectation	
1048.0, *p*<0.001	
Was *more* often followed by:	
Expectation confirmation	<0.001
Expectation violation	<0.001
Monitoring traffic	0.019
Was *not* often followed by:	
Route planning	<0.001
Action planning	<0.001
Coasting	<0.001
Rule related	<0.001
Visual inspection	0.039
Visual inspection	
121.6, *p*<0.001	
Was *more* often followed by:	
Expectation confirmation	0.002
Was *not* often followed by:	
Emotions	<0.001
Expectation confirmation	
243.0, *p*<0.001	
Was *more* often followed by:	
Action planning	<0.001
Monitoring traffic	0.002
Was *not* often followed by:	
Expectation violation	<0.001
Expectation	0.021
Rule related	0.039
Expectation violation	
251.2, *p*<0.001	
Was *more* often followed by:	
Route planning	<0.001
Rule related	0.007
Was *not* often followed by:	
–	
Monitoring traffic	
338.6, *p*<0.001	
Was *more* often followed by:	
Action planning	<0.001
Expectation confirmation	0.001
Was *not* often followed by:	
Emotions	0.001
Visual inspection	0.006
Rule related	
40.3, *p*<0.001	
Was *more* often followed by:	
Action planning	<0.001
Was *not* often followed by:	
Coasting	0.021
Emotions	
45.2, *p*<0.001	
Was *more* often followed by:	
Coasting	0.021
Was *not* often followed by:	
Visual inspection	0.021
Theory of mind	
Not significant	

Degrees of freedom for *χ*^2^ tests=9.

## Appendix A Examples of route planning from the verbal reports

Examples of route planning from the verbal reports are given in the following table:
